# Interdisziplinärer Expertenkonsensus zu Innovationen der bildgebenden Diagnostik und radionuklidbasierten Therapien des fortgeschrittenen Prostatakarzinoms

**DOI:** 10.1007/s00120-021-01598-2

**Published:** 2021-08-18

**Authors:** Dirk Beyersdorff, Kambiz Rahbar, Markus Essler, Ute Ganswindt, Anca﻿-﻿Ligia Grosu, Jürgen E. Gschwend, Kurt Miller, Klemens Scheidhauer, Heinz﻿-﻿Peter Schlemmer, Johannes Maria Wolff, Bernd Joachim Krause

**Affiliations:** 1 Klinik und Poliklinik für Diagnostische und Interventionelle Radiologie und Nuklearmedizin, Universitätsklinikum Hamburg-Eppendorf, Hamburg, Deutschland; 2 Klinik für Nuklearmedizin, Universitätsklinikum Münster, Münster, Deutschland; 3 Klinik und Poliklinik für Nuklearmedizin, Universitätsklinikum Bonn, Bonn, Deutschland; 4 Universitätsklinik für Strahlentherapie und Radioonkologie, Medizinische Universität Innsbruck, Innsbruck, Österreich; 5 Klinik für Strahlenheilkunde, Universitätsklinikum Freiburg, Freiburg, Deutschland; 6 Urologische Klinik und Poliklinik, Klinikum rechts der Isar, München, Deutschland; 7 Klinik für Urologie, Charité – Universitätsmedizin Berlin, Berlin, Deutschland; 8 Nuklearmedizin, Klinikum rechts der Isar, München, Deutschland; 9 Abteilung Radiologie, Universitätsklinikum Heidelberg, Heidelberg, Deutschland; 10 Urologie, Paracelsus-Klinik Golzheim, Düsseldorf, Deutschland; 11 Klinik und Poliklinik für Nuklearmedizin, Universitätsmedizin Rostock, Schillingallee 35, 18057 Rostock, Deutschland

**Keywords:** Prostatakarzinom, Kastrationsresistenz, PSMA-PET/CT, Radium-223, Lutetium-177-PSMA, Prostate cancer, Castration resistance, PSMA-PET/CT, Radium-223, Lutetium-177-PSMA

## Abstract

**Hintergrund:**

Die zahlreichen diagnostischen und therapeutischen Innovationen beim fortgeschrittenen Prostatakarzinom, sowohl in der hormonsensitiven als auch in der kastrationsresistenten Situation, haben in den letzten Jahren zu einer Neuorientierung beim Management dieses Tumors geführt. Ungeachtet der bereits in Teilen in der S3-Leitlinie zu Früherkennung, Diagnose und Therapie des Prostatakarzinoms abgebildeten neuen diagnostischen und therapeutischen Methoden, gibt es in der klinischen Versorgung darüber hinaus gehende Fälle, in denen Patienten von diesen innovativen Verfahren potenziell profitieren könnten.

**Fragestellung:**

Seit Juli 2018 trifft sich deshalb eine interdisziplinäre Expertengruppe aus Nuklearmedizinern, Radiologen, Radioonkologen und Urologen, um ein Konsensuspapier zu Innovationen der bildgebenden Diagnostik und radionuklidbasierten Therapien des fortgeschrittenen Prostatakarzinoms vor dem Hintergrund aktueller Studien und Erfahrungen im klinischen Alltag zu erarbeiten.

**Schlussfolgerung:**

Der Arbeitskreis gibt Anregungen, um zu einer besseren Implementierung neuer bildgebender Techniken, wie multiparametrische Magnetresonanztomographie (mpMRT), PSMA-PET/CT (prostataspezifisches Membranantigen – Positronenemissionstomographie/Computertomographie) und innovativer therapeutischer Optionen (Radium-223-dichlorid, Lutetium-177-PSMA) bei den komplexen Therapieoptionen des fortgeschrittenen Prostatakarzinoms beizutragen.

## Abstract (en)

**Background:**

Numerous diagnostic and therapeutic innovations in the treatment of advanced prostate cancer, both in the hormone-sensitive and in the castration-resistant situation, recently led to a new orientation in the management of this tumor. However, there are potential indications beyond the ones covered by the S3 guideline on early detection, diagnosis and therapy of prostate cancer in clinical care that might be helpful for patients.

**Objectives:**

Since July 2018, an interdisciplinary group of experts from nuclear medicine, radiologists, radio-oncologists and urologists developed a consensus paper on state-of-the-art innovations in imaging diagnostics and radionuclide-based therapies for advanced prostate cancer.

**Conclusions:**

Provided by the working group are suggestions and strategies to improve the implementation of new imaging techniques such as multiparametric magnetic resonance imaging (mpMRI), PSMA-PET/CT (prostate-specific membrane antigen-positron emission tomography/computed tomography) and innovative therapeutic options (radium-223 dichloride, lutetium-177-PSMA) in the complex treatment of metastatic castration-resistant prostate cancer (mCRPC).

## Bildgebende Techniken beim fortgeschrittenen Prostatakarzinom

### Bildgebung bei Primärdiagnose

Wichtigste Methoden im Rahmen der Primärdiagnostik des Prostatakarzinoms sind digital-rektale Untersuchung, transrektaler Ultraschall (TRUS) und Biopsie [1]. Die Sensitivität der Diagnose kann laut Daten einer Phase-III-Studie mit intraindividuellem Vergleich der Methoden durch die MRT im Vergleich zur TRUS-geführten Biopsie verdoppelt werden (93 % vs. 48 %; *p* < 0,0001; [2]). Untermauert wird dieses Ergebnis durch die kürzlich publizierte PRECISION-Studie, in der sich die Risikoabschätzung mittels MRT vor Biopsie und die MRT-gesteuerte Biopsie im Vergleich zur sonographisch gesteuerten Biopsie ebenfalls als überlegen erwies [3]. Die MRT wurde inzwischen differenziert in die S3-Leitlinie aufgenommen und zwar unter bestimmten Voraussetzungen als ergänzende bildgebende Untersuchung, sowohl vor der Erstbiopsie („sollte“), als auch nach negativer Biopsie („soll“) und vor Einschluss in Active Surveillance („soll“; [1]). Es wird explizit erwähnt, dass die MRT multiparametrisch gemäß aktuellen Qualitätsstandards durchgeführt werden „soll“, und dass sie für die initiale Diagnostik, d. h. vor der Erstbiopsie, derzeit noch nicht Teil der leitlinienkonformen Routinediagnostik ist. Die PSMA-PET (prostataspezifisches Membranantigen – Positronenemissionstomographie/Computertomographie) hat eine höhere Genauigkeit für den Nachweis von Prostatakarzinommetastasen als die Kombination von Computertomographie (CT) und Knochenszintigraphie [4]. Die PSMA-PET/CT kann beim High-risk-Prostatakarzinom (Gleason-Score 8–10 oder T‑Kategorie cT3/cT4 oder PSA ≥ 20 ng/ml) zur Ausbreitungsdiagnostik eingesetzt werden [1]. Laut eines aktuellen ASCO-Abstracts verbessert die Kombination der beiden Bildgebungsmodalitäten die Leistung, erreicht jedoch auf Läsionsebene keine statistisch signifikanten Werte und rechtfertigt möglicherweise keine Änderungen der derzeitigen Praktiken [5].

### Primäres Staging

Bildgebende Untersuchungen sind beim primären Staging in Abhängigkeit vom Risikoprofil durchzuführen. Während die S3-Leitlinie bei Patienten mit kleinen Tumoren (cT1) und niedrigem Risiko keine bildgebenden Untersuchungen empfiehlt und bei Patienten mit intermediärem Risiko, aufgrund der unzureichenden Datenlage, keine Empfehlung zur Bildgebung ausspricht, sind derartige Untersuchungen bei höherem Risiko indiziert. So wird bei Patienten mit einem Gleason-Score ≥ 8, einem Tumor der Kategorie cT3/4 oder einem PSA-Wert > 10 ng/ml vor der Therapieentscheidung die Durchführung einer MRT oder CT der Beckenorgane empfohlen [1]. Außerdem wird gemäß S3-Leitlinie bei Verdacht auf einen lokal fortgeschrittenen Tumor (z. B. bei Infiltration der Kapsel und/oder Samenblase) zwecks Festlegung der klinischen T‑Kategorie eine „Kann“-Empfehlung für eine MRT gegeben, sofern diese Untersuchung für die Planung der Strahlentherapie und für die exakte Definition des Zielvolumens notwendig ist. Insbesondere wird das Zielvolumen durch die MRT-basierte Strahlentherapieplanung um ca. 30 % kleiner als bei der Planung mittels CT, wobei sich Prostata und Risikoorgane besser konturieren lassen [6–8]. Patienten mit histologisch gesichertem Tumor und einem PSA-Wert > 10 ng/ml oder einem Gleason-Score ≥ 8 oder einer T‑Kategorie cT3/4 oder Knochenschmerzen sollten leitliniengemäß zudem ein Skelettszintigramm erhalten [1].

Die ligandenbasierte PSMA-Hybridbildgebung wird bislang laut Leitlinie nur innerhalb klinischer Studien empfohlen. Daten retrospektiver, einarmiger Studien weisen darauf hin, dass diese neue Technik bei Patienten mit intermediärem bis hohem Risiko zu einer Steigerung der Sensitivität um 30 % und der Spezifität auf nahe 100 % im Vergleich zur herkömmlichen CT oder MRT führt [9–11].

In einer prospektiven Arbeit von Van Leeuwen et al. wurde gezeigt, dass die Durchführung eines PSMA-PET/CT vor einer primären Strahlentherapie das Therapiekonzept bei 21 % der Patienten verändert [12]. Das PSMA-PET/CT kann befallene Lymphknoten im Becken und eine eventuelle Metastasierung visualisieren. Dadurch kann der Lymphabfluss je nach Befall im Zielvolumen erfasst oder nicht erfasst werden – eine Entscheidung, die einen relevanten Einfluss auf die Ergebnisse der Strahlentherapie hat. Des Weiteren kann das PSMA-PET/CT das GTV („gross tumor volume“) in der Prostata darstellen und dadurch einer fokalen Dosiseskalation dienen [13, 14].

Um die PSMA-basierte PET-Hybridbildgebung in die breite Versorgung zu bringen, bedarf es nach Ansicht der Expertengruppe jedoch weiterer kontrollierter Studien. Neue Erkenntnisse wird eine derzeit laufende, vom Deutschen Konsortium für translationale Krebsforschungszentrum (DKTK) durchgeführte Multicenterstudie zum prospektiven Vergleich von PSMA-PET/CT vs. Histopathologie liefern, für die Patienten mit geplanter Prostatektomie und erweiterter Lymphadenektomie im Rahmen der Initialdiagnostik rekrutiert werden (klinische Prüfung der Phase I/II; ClinicalTrials.gov identifyer NCT03362359; Ga-68-PSMA-11 in „high-risk prostate cancer“).

### Diagnostik bei PSA-Rezidiv

Bei Patienten mit PSA-Rezidiv nach Primärtherapie empfiehlt die S3-Leitlinie, anhand klinischer Faktoren (z. B. PSA-Verdopplungszeit) zwischen lokalem und systemischem Rezidiv zu differenzieren [1]. Allerdings beurteilt die Expertengruppe diese Faktoren mittlerweile als zu wenig aussagekräftig. Immerhin wird in der aktualisierten S3-Leitlinie die PSMA-basierte PET-Hybridbildgebung bereits als „Kann“-Empfehlung zur Beurteilung der genauen Tumorausdehnung aufgeführt, sofern der Befund therapeutische Konsequenzen hat [1]. Der Beginn einer frühen Rezidivtherapie soll durch den Einsatz dieser neuen Technik nicht verzögert werden. Künftig wird das PSMA-PET an Bedeutung gewinnen, da es selbst in einem sehr niedrigen PSA-Bereich von 0,2–0,5 ng/ml die Erkennung von Rezidiven ermöglicht. Die PSMA-basierte PET kann damit bereits sehr früh Hinweise auf ein Lokalrezidiv und eine eventuelle Lymphknoten- oder Knochenmetastasierung liefern und so ein frühzeitiges therapeutisches Eingreifen einerseits, aber auch eine Änderung der Therapiestrategie andererseits bewirken (z. B. operative Lymphknotenresektion oder Strahlentherapie bei Oligometastasen). PET-CT und PET-MRT sind hinsichtlich ihrer Sensitivität als gleichwertig einzuschätzen [10]. Weitere Daten zur PSMA-basierten PET-Bildgebung beim Staging und Restaging liefern zwei neuere systematische Reviews und Metaanalysen [15, 16]. Perera et al. zeigten, dass Sensitivität und Spezifität dieser Technik mit zunehmendem PSA-Spiegel deutlich ansteigen und die Befunde bei Werten > 2 ng/ml nahezu immer positiv ausfallen [15].

Für die Strahlentherapie kann das PSMA-PET insofern einen entscheidenden Zugewinn an Informationen bringen und zu einer Änderung des Therapiemanagements führen, da bei Vorliegen einer lokalisierten bzw. metastasierten Erkrankung unterschiedliche Therapiekonzepte zu verfolgen sind [17, 18]. Auch die exakte Tumorlokalisation ist für die Bestrahlungsplanung und eine eventuelle fokale Dosiseskalation bei gleichzeitiger Schonung von Rektum und Blase von entscheidender Bedeutung [19].

### Derzeitige Themen in Bezug auf die PSMA-basierte Bildgebung in Deutschland

Bislang ist in Deutschland kein PSMA-Radiopharmakon zugelassen. Die Kostenerstattung für diese bildgebende Technik ist regional sehr heterogen und nicht einheitlich geregelt. Hier sind weitere gemeinsame Schritte aller beteiligten Interessengruppen notwendig, um perspektivisch die PET/CT in die onkologische Regelversorgung zu implementieren.

Um die Voraussetzungen für die Zulassung zu schaffen, fordert die Expertengruppe sowohl zulassungsrelevante Studien in Kooperation mit industriellen Partnern, als auch durch öffentliche Förderung auf nationaler Ebene. Kontrovers diskutiert wurden patientenrelevante Endpunkte: Ein Teil der Experten erachtet Sensitivität/Spezifität bzw. Änderung des therapeutischen Vorgehens als sinnvolle Endpunkte. Andere Experten gaben zu bedenken, dass die Auswirkungen auf das Gesamtüberleben und andere patientenrelevante Endpunkte durch die neue Bildgebung bisher nicht gesichert ist. Dabei sind Anforderungen an Endpunkte zwischen diagnostischen und therapeutischen Studien zu diskutieren.

## Therapie mit Radium-223-dichlorid beim mCRPC

Die Phase-III-Studie ALSYMPCA schloss 921 CRPC-Patienten mit symptomatischen Knochenmetastasen (≥ 2 in der Skelettszintigraphie) ohne bekannte viszerale Metastasen (CT/MRT Abdomen/Becken und CT/Röntgen Thorax) ein, die randomisiert im Verhältnis 2:1 zusätzlich zur bestmöglichen Standardtherapie Radium-223-dichlorid (Radium-223; 6 × 1 Injektion im 4‑Wochen-Intervall) oder Placebo erhielten [20]. Die aktive Therapie mit dem Alpha-Strahler führte zu einer signifikanten Verlängerung des Gesamtüberlebens (primärer Endpunkt) um 3,6 Monate im Vergleich zu Placebo (14,9 vs. 11,3 Monate; Hazard Ratio [HR] 0,70; *p* < 0,001). Der Überlebensvorteil von Radium-223 galt für alle analysierten Subgruppen und war unabhängig von einer Docetaxel-Vortherapie. Weiterhin wurde die Zeit bis zum Auftreten des ersten symptomatischen skelettalen Ereignisses (pathologische Fraktur, Rückenmarkkompression, externe Bestrahlung zur lokalen Schmerzkontrolle, orthopädische Intervention) gegenüber Placebo signifikant um 5,8 Monate verlängert (15,6 vs. 9,8 Monate; *p* < 0,001). Die Therapie mit Radium-223 zeichnete sich durch ein günstiges Verträglichkeitsprofil aus [18]. Auf Basis der ALSYMCPA-Studie wurde Radium-223 von der EMA für mCRPC-Patienten mit symptomatischen Knochenmetastasen zugelassen.

Im Juni 2018 hat die EMA die Indikation für Radium-223 eingeschränkt [21]. Grundlage dafür sind die Ergebnisse der Phase-III-Studie ERA-223. In dieser Studie wurde die Kombinationstherapie Abirateron/Radium-223 gegen Abirateron bei Patienten mit nicht- oder mild-symptomatischem mCRPC verglichen [22]. 806 Patienten wurden 1:1 randomisiert. Primärer Endpunkt war das symptomatische, skelettale, eventfreie Überleben (SSE-FS). Die Studie wurde vorzeitig entblindet, da im Kombinationsarm mehr Frakturen und Todesfälle beobachtet wurden.

Das mediane SSE-FS betrug 22,3 Monate (95 %-Konfidenzintervall [‑KI] 20,4–24,8) im Radium-223-/Abirateron-Arm und 26,0 Monate (95 %-KI 21,8–28,3) im Abirateron‑/Placeboarm (HR 1,122; 95 %-KI 0,917–1,374; *p* = 0,2636). Frakturen traten bei 112/392 (29 %) Patienten im Abirateron‑/Radium-223-Arm und 45/394 (11 %) Patienten im Abirateron‑/Placeboarm auf. In Post-hoc-Analysen war die Verwendung von knochenprotektiven Substanzen (Denosumab, Zoledronsäure) bei Beginn der Studie in beiden Armen mit niedrigeren Frakturraten verbunden. Das mediane Gesamtüberleben betrug 30,7 Monate (95 %-KI 25,8 – „not estimable“ [NE]) im Abirateron‑/Radium-223-Arm und 33,3 Monate (95 %-KI 30,2–41,1) im Abirateron‑/Placeboarm (HR 1,195; 95 %-KI 0,950–1,505; *p* = 0,1280). Die Kombination aus Abirateron und Radium-223 war damit Abirateron als Monotherapie beim primären Endpunkt (SSE-FS) nicht überlegen, der Unterschied beim Gesamtüberleben war nicht signifikant. Die im Kombinationsarm vermehrt aufgetretenen Frakturen waren nicht in den Bereichen, in denen Metastasen vorlagen; die genaue Genese ist unklar – am wahrscheinlichsten liegen durch Osteoporose bedingte zusätzliche Frakturen vor.

Die EMA hat nach Veröffentlichung der Studienergebnisse Indikationseinschränkungen für die Verwendung von Radium-223 beim mCRPC beschlossen [21], die hier zusammengefasst dargestellt werden:

- Radium-223-dichlorid sollte nur als Monotherapie oder in Kombination mit einem LHRH-Analogon bei Patienten mit mCRPC, symptomatischen Knochenmetastasen und keinen nachgewiesenen viszeralen Metastasen eingesetzt werden, die nach mindestens zwei Linien mCRPC-Therapie progredient sind, oder für eine andere verfügbare systemische mCRPC-Therapie nicht geeignet sind.
- Radium-223-dichlorid ist in der Kombination mit Abirateron kontraindiziert.
- Radium-223-dichlorid wird nicht empfohlen bei Patienten mit einem niedrigen Level an osteoblastischen Metastasen und bei Patienten mit asymptomatischen Knochenmetastasen.
- Bei mild symptomatischen Patienten ist der Nutzen der Behandlung sorgfältig gegen die Risiken abzuwägen.
- Vor und während der Behandlung mit Radium-223-dichlorid sollte eine Beurteilung der Knochendichte per Dexa-Scan erfolgen.
- Die gleichzeitige Behandlung mit Denosumab oder Zoledronsäure sollte in Betracht gezogen werden.

Die meisten von der EMA empfohlenen Einschränkungen bei der Monotherapie mit Radium-223 sind durch die Daten der ERA-223-Studie nicht abgedeckt und eher als Vorsichtsmaßnahme zu werten. Von der FDA und der japanischen Zulassungsbehörde wurden keine Einschränkungen zur Monotherapie empfohlen. Die Indikationsstellung auf Basis der ALSYMPCA-Daten ist durch die ERA-223-Ergebnisse inhaltlich nicht berührt, Radium-223 sollte jedoch nicht mehr in der Kombination mit Abirateron gegeben werden. Eine Radium-223-Therapie beim mCRPC ist innerhalb der neuen EMA-Empfehlungen nach zwei Hormonbehandlungen (Abirateron und Enzalutamid), nach Hormonbehandlung und Chemotherapie, oder wenn der Patient für keine der anderen systemischen Therapien geeignet ist, möglich.

Für die praktische Durchführung der Behandlung mit Radium-223 zum Therapiemonitoring sollte die gesamtalkalische Phosphatase (tALP) herangezogen werden [23]. Eine exploratorische Analyse der ALSYMPCA-Studie zeigte, dass zu Woche 12 87 % der Radium-223-Patienten einen Abfall der tALP hatten (Placebopatienten: 23 %; *p* < 0,0001). Die klinische Relevanz dieser Verringerung zeigte sich dadurch, dass ein Abfall der tALP dabei stark mit einem verlängerten Gesamtüberleben korrelierte: Patienten der Radium-223-Gruppe mit nachweislichem tALP-Abfall hatten im Vergleich zu Patienten ohne tALP-Reduktion ein um 7,4 Monate verlängertes Gesamtüberleben (17,8 vs. 10,4 Monate; HR 0,45; [23]).

Zur Durchführung der Radionuklidtherapie von Knochenmetastasen mit Radium-223 gibt eine DGN-Handlungsempfehlung von 2016 detaillierte Hinweise, welche allerdings unter dem Hintergrund der aktuellen Indikationsstellung zu bewerten sind [24].

Radium-223 als Monotherapie sollte nach Ansicht der Experten auch weiterhin zur Behandlung von Patienten mit symptomatischen Knochenmetastasen empfohlen werden. Die Experten weisen darauf hin, dass die Symptomatik als Kontinuum zu betrachten ist und zwischen schwach ausgeprägten und stärkeren Beschwerden nicht exakt differenziert werden kann. Bei stark schmerzenden und/oder fraktur-/kompressionsgefährdeten Knochenmetastasen und hohem skelettbedingtem Komplikationsrisiko ist prinzipiell eher eine lokale perkutane Strahlentherapie indiziert. Neue Konzepte sehen bei skelettaler Oligometastasierung eine kleinvolumige Radiatio in höherer Strahlendosis mit Konzentration auf die Tumormasse vor, um eine maximale lokale Tumorkontrolle zu erreichen. Zusätzlich kann Radium-223 in Kombination mit lokaler Bestrahlung oder sequenziell verabreicht werden; ggf. sind auch operative Interventionen angezeigt [1].

## Therapie mit Lu-177-PSMA beim mCRPC

Die Radioligandentherapie (RLT) mit Lutetium-177-markiertem PSMA (Lu-177-PSMA-Therapie) wurde klinisch maßgeblich in Deutschland entwickelt. Im Dezember 2015 wurde im Rahmen einer Expertenrunde der DGN eine Konsensusempfehlung für die Therapie mit Lu-177-PSMA-617 erarbeitet, die Orientierung für die Durchführung dieser RLT geben soll [25]. Die Konsensusempfehlung enthält Empfehlungen zu Indikationsstellung, Voruntersuchung, Therapieablauf/Begleittherapie, Dosimetrie und Nachsorge. Die Lu-177-PSMA-Therapie kann Patienten, die gemäß S3-Leitlinie alle zugelassenen Therapien erhalten haben und dennoch progredient sind, in *Ausübung ärztlicher Heilkunde *und auf Basis einer interdisziplinären Tumorkonferenz angeboten werden [1].

In der deutschen Multicenterstudie zur Lu-177-PSMA-617-Therapie wurden Daten von 145 Patienten mit metastasiertem kastrationsresistenten Prostatakarzinom (mCRPC), die 1–4 Zyklen Lu-177-PSMA-Therapie erhielten, retrospektiv ausgewertet. Insgesamt wurden 248 Therapiezyklen bei den 145 Patienten durchgeführt [26]. Das gesamte biochemische Ansprechen lag bei 45 % nach allen Therapiezyklen. Grad-3- bis -4-Hämatotoxizität trat bei 18 Patienten auf. Nebenwirkungen waren Anämie (10 %), Thrombozytopenie (4 %) und Leukopenie (3 %). Eine Xerostomie trat bei 8 % der Patienten auf [26]. In einem Review wurde von Emmett et al. eine Übersicht der bis 2017 veröffentlichten retrospektiven klinischen Studien publiziert und im Hinblick auf das Therapieansprechen, Toxizität und Sicherheit diskutiert [27].

Inzwischen liegen zwei systematische Reviews und die Publikation einer unizentrischen Phase-II-Studie aus Australien vor [28–30]. Calopedos et al. publizierten einen systematischen Review und Metaanalyse, in die 10 Studien mit 369 Patienten (davon 334 analysierbar) eingeschlossen wurden [28]. Der gepoolte Anteil mit einem PSA-Abfall jedweder Größenordnung betrug 68 %, mit einem PSA-Abfall > 50 % 37 %. Von Eyben et al. schlossen in ihren systematischen Review 12 Studien mit 669 Patienten ein. Der gepoolte Anteil mit einem PSA-Abfall > 50 % betrug 43 % [29].

Prospektive Daten randomisierter kontrollierter Studien zur Lu-177-PSMA-Therapie liegen derzeit nur für 2 Phase-II-Studien aus Australien vor [30, 31]: In einer einarmigen unizentrischen Phase-II-Studie wurden 50 Patienten eingeschlossen und mit bis zu 4 Zyklen Lu-177-PSMA-617 therapiert. Ein PSA-Abfall > 50 % wurde bei 32 der 50 Patienten (64 %) beobachtet; davon lag bei 22 Patienten (44 %) der PSA-Abfall sogar bei > 80 %. Es gab keine therapieassoziierten Todesfälle. Die häufigsten Nebenwirkungen waren Mundtrockenheit, Übelkeit, Fatigue und Thrombozytopenie [30].

Des Weiteren liegen seit Anfang 2021 die Daten der TheraP-Studie, eine multizentrische, nicht verblindete, randomisierte Phase-II-Studie an 11 Zentren in Australien vor. Hier wurden 291 Probanden 1:1 auf Lu-177-PSMA-617 (6,0–8,5 GBq i.v. alle 6 Wochen für bis zu 6 Zyklen) oder Cabazitaxel (20 mg/m^2^ i.v. alle 3 Wochen für bis 10 Zyklen) randomisiert. Ein PSA-Abfall von > 50 % war bei Patienten in der Lu-177-PSMA-617-Gruppe häufiger als in der Cabazitaxel-Gruppe (65 vs. 37 PSA-Antworten; 66 % vs. 37 % bei Behandlungsabsicht; Unterschied 29 %; 95 %-KI 16–42; *p* < 0,0001; und 66 % gegenüber 44 % nach erhaltener Behandlung; Differenz 23 % [9–37]; *p* = 0,0016). Nebenwirkungen der Schwere 3–4 traten bei 32 (33 %) von 98 Männern in der Lu-PSMA-617-Gruppe gegenüber 45 (53 %) von 85 Männern in der Cabazitaxel-Gruppe auf [31]. Diese Daten haben sicherlich entsprechende Konsequenzen auf die Wahl der Therapiesequenz.

Aktuell ist bei einer internationalen, prospektiven, unverblindeten, multizentrischen und randomisierten Phase-III-Studie zur Lu-177-PSMA-Therapie die Rekrutierung abgeschlossen und die Ergebnisse werden zeitnah erwartet (VISION: An international, prospective, open label, multicenter, randomized phase III study of 177Lu-PSMA-617 in progressive PSMA positive metastatic castration resistant prostate cancer [mCRPC]; https://clinicaltrials.gov/ct2/show/NCT03511664; [32]).

Die Teilnehmer des Expertenmeetings waren sich einig, dass potenziell positive Ergebnisse einer prospektiven kontrollierten Phase-III-Studie mit dem Endpunkt Gesamtüberleben unverzichtbare Voraussetzung für einen breiteren Einsatz der 177-Lu-PSMA Therapie im Rahmen eines multimodalen Therapiekonzepts beim mCRPC nach entsprechender Zulassung wären.

In der S3-Leitlinie ist die Lu-177-PSMA-Therapie bei metastasierten CRPC-Patienten in gutem Allgemeinzustand (AZ) erst nach Ausschöpfen aller empfohlenen und zugelassenen Therapieoptionen als Therapieversuch aufgeführt [1].

## Perspektiven

Die neue PSMA-basierte Hybridbildgebung ermöglicht nach bisheriger Datenlage eine deutliche Verbesserung der Diagnostik im Rahmen des PSA-Rezidivs. Nicht ausreichende Daten aus prospektiven Studien stehen bisher einer weiteren Verbreitung (z. B. in beim Primärstaging) entgegen.

Für die Planung der Strahlentherapie könnte das PSMA-PET/CT bei der primär definitiven Strahlentherapie (perkutane Bestrahlung oder Brachytherapie) eine wichtige Rolle einnehmen, da so insbesondere befallene Lymphknoten besser detektiert und Fernmetastasen ausgeschlossen werden können. Die Strahlentherapiefelder ändern sich z. B. nach PSMA-PET/CT deutlich bei bis zu 30 % der Patienten [33]. Dies gilt analog auch für die operative Therapie. Hier konnte in retrospektiven und einer prospektiven Studie gezeigt werden, dass sich das operative Vorgehen bei in der PSMA-PET/CT suspizierten Lymphknotenmetastasen mit einer Erweiterung der Lymphadenektomie oder bei Nachweis von Fernmetastasen gänzlich ändern kann [4, 18]. In einer prospektiven randomisierten Phase-III-Studie hatte das PSMA-PET/CT bei mehr als der Hälfte der Patienten einen Einfluss auf den SRT-Plan. Das Langzeit-Follow-up wird jedoch erst zeigen, ob die Auswirkungen von PSMA-PET auf die SRT-Planung zu einem verbesserten Ergebnis führen oder nicht [34].

In der Rezidivsituation (PSA-Rezidiv) nach lokaler Primärtherapie (radikale Prostatektomie, Strahlentherapie) dient das PSMA-PET/CT zur Differenzierung zwischen Lokalrezidiv, Lymphknotenmetastasen und Knochenmetastasen. Damit können einerseits Patienten identifiziert werden, die für eine potenzielle kurative Therapie infrage kommen bzw. andererseits Patienten, die eher eine antihormonelle oder andere systemische Therapie erhalten sollten. Im Hinblick auf die Therapieplanung beantwortet das PSMA-PET/CT also wichtige Fragen und hat damit entscheidenden Einfluss auf die Behandlungsstrategie [1].

Die systemische Radionuklidtherapie mit Radium-223-dichlorid und radioaktiv markierten PSMA-Analoga hat zu einer deutlichen Erweiterung der Therapieoptionen beim mCRPC geführt. Perspektivisch könnte ein früherer Einsatz von Radium-223-dichlorid vorteilhaft sein, um eine längerfristige Kontrolle von Knochenmetastasen zu ermöglichen.

Die vielversprechenden Daten zu Lu-177-PSMA müssen durch prospektive randomisierte Studien bestätigt werden. Neue Radionuklide wie Actinium-225 und Thorium-227 werden derzeit klinisch bzw. präklinisch erforscht.
